# The influence of different types of fixed orthodontic appliance on the growth and adherence of microorganisms (in vitro study)

**DOI:** 10.4317/jced.50988

**Published:** 2013-02-01

**Authors:** Hayder F. Saloom, Harraa S. Mohammed-Salih, Shaymaa F. Rasheed

**Affiliations:** 1 BDS, MSc. Assistant Profesor of Orthodontics. Department of Orthodontics, College of Dentistry, Baghdad University; 2 BDS, MSc. Assistant lecturer of Orthodontics. Department of Orthodontics, College of Dentistry, Baghdad University; 3 BSc, MSc. Assistant Lecturer of Microbiology. Department of Biology, College of Science, Baghdad University

## Abstract

Orthodontic appliances serve as different impact zones and modify microbial adherence and colonization, acting as foreign reserves and possible sources of infection. This study was conducted to investigate the effect of different types of fixed orthodontic appliances on the growth and adherence of microorganisms in oral flora which are Streptococcus mutans (S. mutans) and Candida albicans. Sixty-four of four different fixed orthodontic appliance-samples were used, divided into four groups of sixteen. Type I: Sapphire brackets- Coated wires, type II: Sapphire brackets- Stainless steel wires, type III: Stainless steel brackets- Coated wires and type IV: Stainless steel brackets- Stainless steel wires. Oral strains of S. mutans and Candida albicans were studied in the present study using biochemical test then microbial suspensions were prepared to do the tests of each microorganism including the antimicrobial effects of different appliance-samples on the growth of microorganisms and their adhesion tests. The results showed significant differences between the different appliances in terms of inhibition zone formation (P<0.001). The adhesion test, which is classified into low, medium and high, showed the adhesion of S. mutans, is low with type I and II, medium with type III and high with type IV, whereas the adhesion of Candida albicans is medium with both type I and II and high with both type III and IV with high significant differences (P<0.001). Appliance with high esthetic appearance, sapphire brackets and coated arch wire, showed the least adherence of S. mutans and Candida albicans in comparison to other appliances with less esthetic and more metal components.

** Key words:**Orthodontic appliance, Adherence, Streptococcus mutans, Candida albicans.

## Introduction

For a long time, the traditional orthodontic patient was considered as a low-risk patient and orthodontic proce-dures were considered noninvasive (1). However, these appliances can be associated to difficulty in cleaning. During treatment, retentive areas are created that favor biofilm accumulation and bacterial growth (2). One of the greatest challenges in orthodontics consists in maintaining proper oral hygiene during treatment. Brackets, bands and other accessories further aggravate these conditions by retaining dental plaque, which can lead to gingivitis and enamel demineralization, causing white spots and caries (3). Microbiological studies have established that, after placement of a fixed orthodontic appliance, the number of bacteria raises significantly, particularly streptococci and lactobacilli, subjecting the oral environment to an imbalance and enabling the emergence of diseases. Although dental biofilm is composed of numerous species of bacteria, it is believed that S. mutans is involved in the early development of carious lesions (4). Thus, orthodontic treatment success lies in correcting occlusion in the best possible manner without, however, affecting the preexisting health of teeth and supporting tissues. Otherwise, treatment benefits may be questioned (5). Orthodontic practice undergoes constant progress with the use of new techniques and materials that benefit both patients and practitioners (6). Attempts to inhibit the development of carious lesions in orthodontic patients have been focused on controlling the bacterial biofilm around the brackets (3). During therapy, orthodontists are also responsible for caries prevention (5).

Orthodontic appliance serves as different loci for biofilm formation (7). In a study by Eliades et al. (8) stainless steel presented the highest critical surface tension and can be expected to have a higher plaque retaining capacity. Metallic orthodontic brackets have been found to induce specific changes in the oral environment such as reduced levels of PH and affinity of bacteria to a metallic surface because of electrostatic reactions (9), also it increased plaque accumulation, and elevated S. mutans colonization. Nevertheless other studies on possible differences in the initial affinity and adherence of bacteria on metal, ceramic, and plastic brackets over time were inconclusive (10,11). Therefore, it is difficult to make a clear assessment that metal brackets have a lower cariogenic effect on the teeth than plastic or ceramic brackets.

The insertion of orthodontic wire tends to create new surfaces available for plaque formation and therefore to increase the level of microorganisms in the oral cavity. It has long been suggested that orthodontic bands and wires lead to an increased plaque accumulation and elevated levels of streptococci and lactobacilli. In addition, orthodontic patients with fixed appliances frequently present an abundance of *S. mutans* in plaque compared with untreated orthodontic patients (12). Therefore, prevention of bacterial attachment to orthodontic wires is a critical concern for orthodontists.

Yeast of the *Candida* genus, *albicans* species, was analyzed because it is the most frequently found microorga-nism in infections of the buccal mucosa. This yeast has been proven to colonize on cement, enamel, and dentin, which serve as a reservoir for the spread of infection (13). Nevertheless, the yeast’s ability to survive on inert surfaces needs to be further elucidated in order to understand its virulence and dissemination routes (14). *S. mutans* was studied because of its well-documented role in the pathogenesis of caries (15-17).

Although a number of studies have demonstrated the viability of* Candida albicans* and *S. mutans* on removable orthopedic appliances, little is known about their survival on fixed orthodontic appliances (18). Also nowadays with the advent of increased orthodontic treatment for adult patients, especially females, and the use of esthetic brackets and/or arch wires has become increasingly popular, bringing about the need to address questions regarding microorganism adherence and biofilm development; therefore this study aimed to investigate the influence of different types of fixed orthodontic appliances on the adherence of most effective microorganisms.

## Material and Methods

- Experimental Groups and Microorganisms 

Sixty- four of four different fixed orthodontic appliance-samples were used in the present study. Experimental groups were divided as follows: Group I: Sapphire brackets- Coated wires (n =8), Group II: Sapphire brackets- Stainless steel wires (n= 8), Group III: Stainless steel brackets- Coated wires (n=8) and Group IV: Stainless steel brackets- Stainless steel wires (n=8). All the brackets used were for the right upper first premolar with a 0.022 slot with hooks for the straight-arch technique. All the arch wires used were 0.016x0.022 straight arch wires.

Each type of brackets was ligated with 10mm of wire using elastic ligature. The procedure was performed under same condition using sterile straight point tweezers (Bracket holder) with sterile artery forceps.

The following strains were studied: oral strains of *S. mutans and Candida albicans. S. mutans* was seeded in Todd Hewitt broth under anaerobic conditions at 37C° for 24 hours (hr).* Candida albicans* was seeded in Sabo-reau dextrose agar and incubated under aerobic conditions at 37C° for 18 hr. Oral strains of *S. mutans and Can-dida albicans* identified using biochemical test then microbial suspensions were prepared to do the tests of each microorganism.

- Anti-Microbial Effects of Fixed Orthodontic Appliances on Growth of Microorganisms

This test was used to detect the anti-microbial effect of different fixed orthodontic appliance- samples on growth of *S. mutans and Candida albicans* (in vitro). Brain heart infusion agar was used in this test, 8 plates for each microorganisms, inoculated with microbial suspension *S. mutans and Candida albicans* by using swabbing, then different types of appliance- samples (4 samples from each group) were placed in each Brain heart infusion agar plate with a distance of 50mm between the four appliance-samples to allow enough space for each sample to reflect its anti-microbial effect on growth of microorganism. After that all plates incubated at 37 C° and examined after 24 hr and 48 hr. Inhibition zones formed were measured in millimeter by using the digital vernier.

- Adhesion Tests

This test was done by using glass test tubes. It was used to detect the ability of *S. mutans and Candida albicans* to adhere to smooth surface (glass test tubes) and to detect the influence of different Fixed Orthodontic appliance- samples on adherence of microorganisms (in vitro).

This test was done by adding 5ml of tryptic soy broth contain 20% sucrose for 8 tubes for each microorganism in each experimental group. This medium was used given that mutans streptococci produce glucans from sucrose which allows adherence to the glass surface of the test tube. Then each tube was inoculating with 0.5ml of microbial suspension *S. mutans and Candida albicans*, after that the appliance-samples were transferred to each tubes. The control tube contain only medium without microbial suspension. The tubes were tilted at an angle of 25° and incubated under microanaerobiosis at 37C° for 2 days. After incubation, periodontents medium of each tube were removed and tubes were stained by adding 5ml of 0.1% safranin for 1 minute then the contents of tubes were removed. Macroscopic observation of the samples was performed and the results were read as following:

Adherence was scored as low, medium, or high. Adherence was considered low when affine biofilm was obser-ved on the wall of the test tube and found to detach completely on setting the tube upright. Partial detachment of the biofilm was considered medium adherence, and no detachment was considered as high adherence (1).

## Statistical Analysis

The results were expressed by measuring the inhibition zone area formed around each appliance-sample in each group in mm and the results of adhesion tests were expressed in non-numerical parameters. Statistical analyses were performed including means and standard errors of the mean for each group. One-way analysis of variance (ANOVA) with t- test was used for multiple comparisons. A value of P<0.05 was considered significant.

## Results

- Anti-Microbial Effect Test

The results obtained under these experimental conditions showed that the capacity of *S. mutans and Candida albicans* to adhere to four different fixed orthodontic appliance-samples was determined.

S. mutans

 Table 1 shows the inhibition zone formed around the four different orthodontic appliance- samples after 24 hr and 48 hr, which showed that the largest inhibition zone was found with type I then type II followed by type III and the smallest with type IV in both testing time.

**Table 1:**
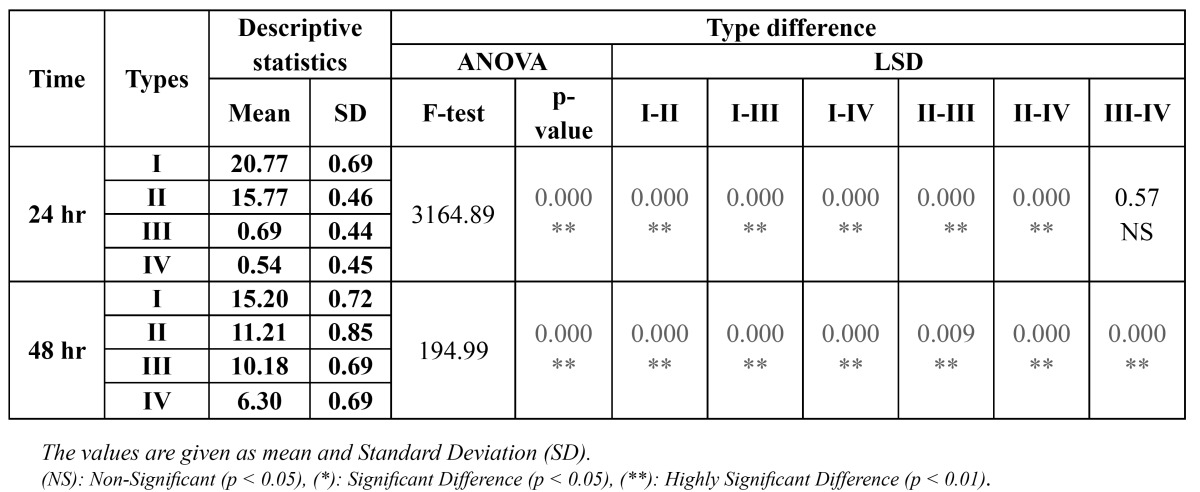
Inhibition zone formed with S. mutans using different types of fixed orthodontic appliance.

To compare the inhibition zone among four types, ANOVA test was used and showed a high significant difference with all types (P<0.001). Least significant difference test (LSD test) was used to compare between each two types showed a high significant difference (P<0.01) except between type III and IV showed no significant difference (P>0.05) after 24 hr ( Table 1).

To compare between the inhibition zone formed after 48 hr around each type, t-test was used and showed a high significant reduction in the inhibition zone with type I and II and a high significant increment with type III and IV (P<0.001) in comparison to those after 24 hr, ( Table 2).

**Table 2:**
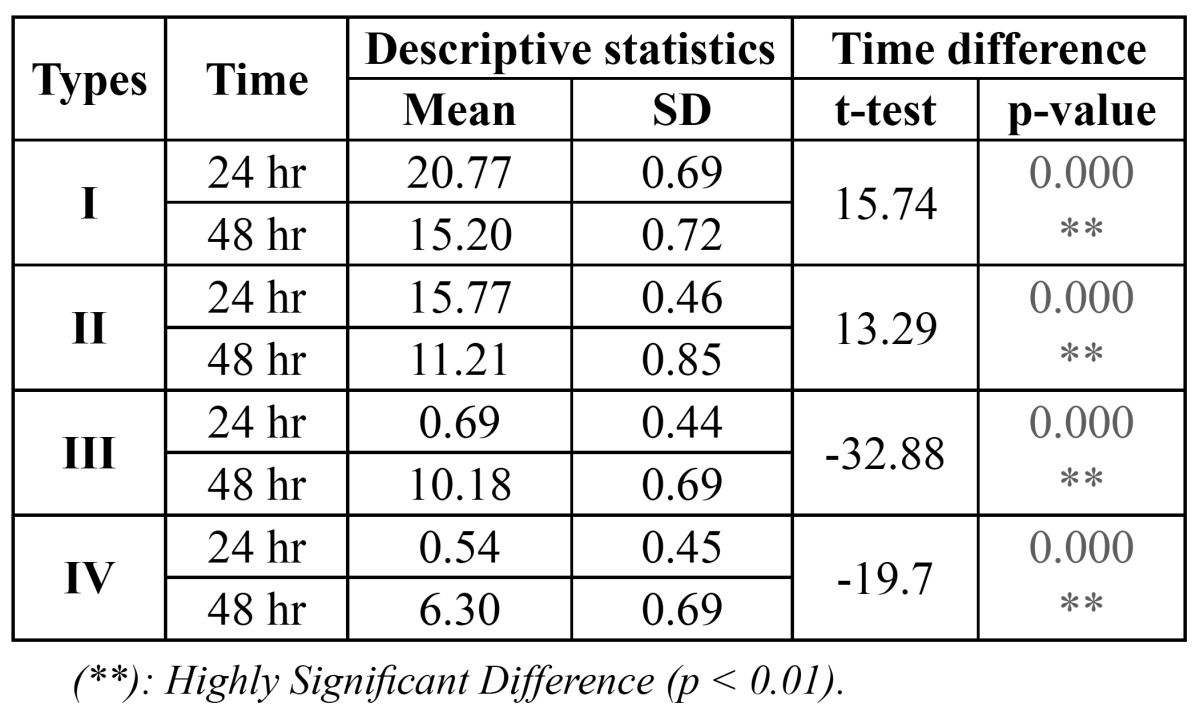
Time difference in the inhibition zone formed with S. mutans using different types of fixed orthodontic appliance.

Candida albicans

The inhibition zone formed by *Candida albicans*, after 24 hr, is largest with type I then type II followed by type III and smallest with type IV, whereas after 48 hr, the largest inhibition zone occurred with type I, then type III followed by type II and the smallest with type IV, ( Table 3).

**Table 3:**
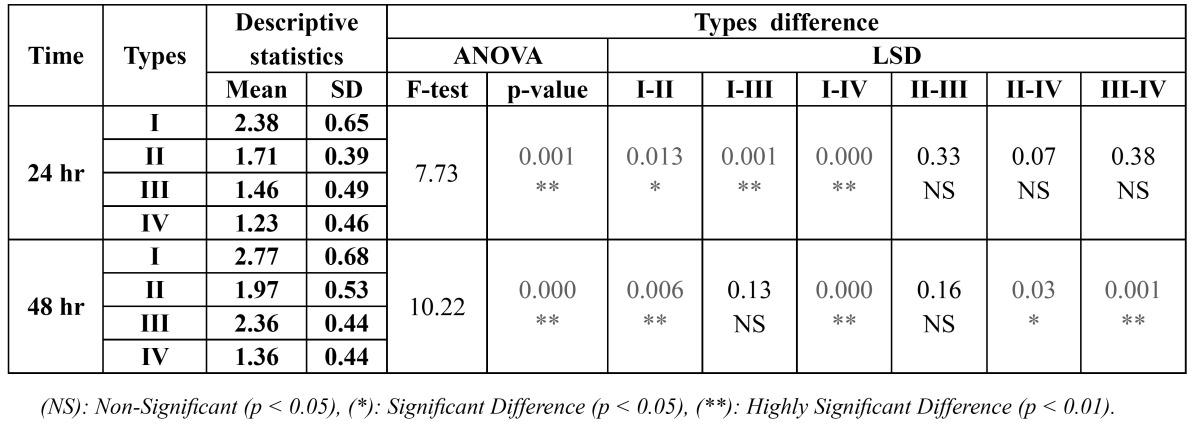
Inhibition zone formed with Candida albicans using different types of fixed orthodontic appliance.

Comparison among four different types of fixed orthodontic appliance was done using ANOVA test which showed a high significant difference (P<0.01) with all types and in both testing time. LSD- test was done to compare between each two groups and reveal that, after 24 hr, type I differs significantly from type II (P<0.05) and highly significantly from type III and IV. Type II differs significantly from type I (P<0.05) and insignifi-cantly (P>0.05) from type III and IV. Type III and IV show a high significant difference (P<0.01) with type I and no significant difference with the others (P>0.05). After 48 hr, LSD test show no significant difference (P>0.05) between type I and III, and between type II and III. Significant difference (P<0.05) was found between type II and IV, and a high significant difference (P<0.01) between the remaining groups has been noticed.

For time difference, t-test was applied and showed that the inhibition zones formed by the *Candida albicans* after 48 hr are insignificantly larger (P>0.05) than those formed after 24 hr with all appliances type except with type III appliance which is statistically significant (P<0.05), ( Table 4).

**Table 4:**
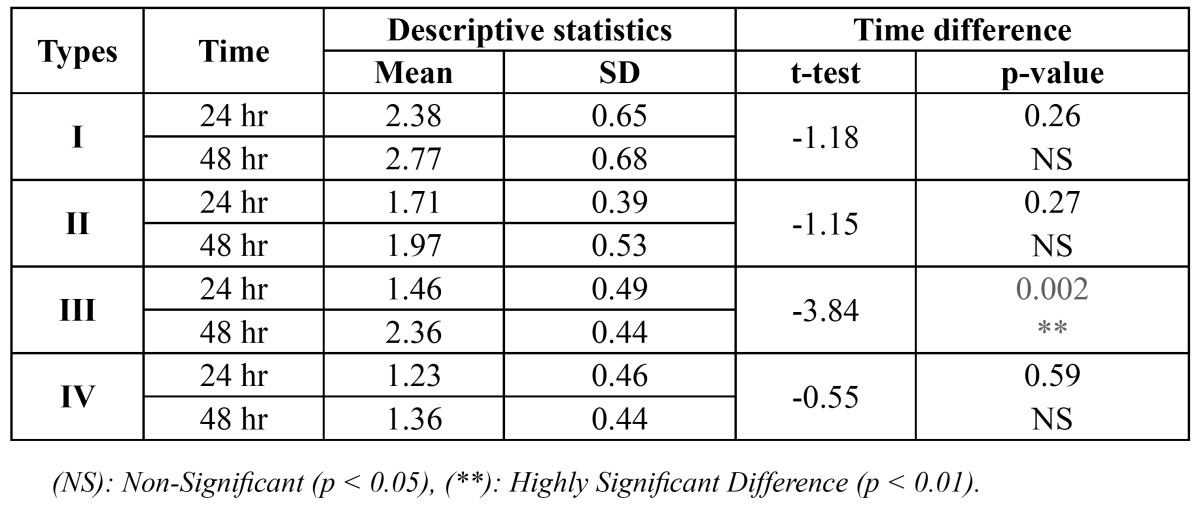
Time difference in the inhibition zone formed with Candida albicans using different types of fixed orthodontic appliance.

- Adhesion Test 

**Figure 1:**
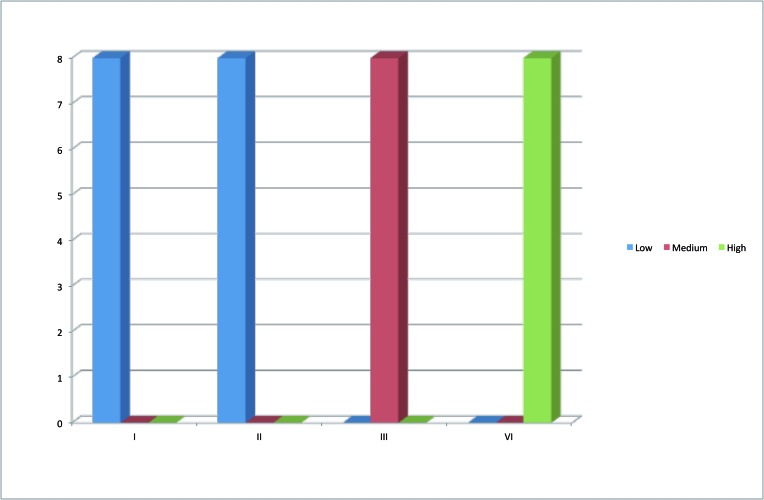
Adhesion test of S. mutans with different types of orthodontic appliance.

**Figure 2:**
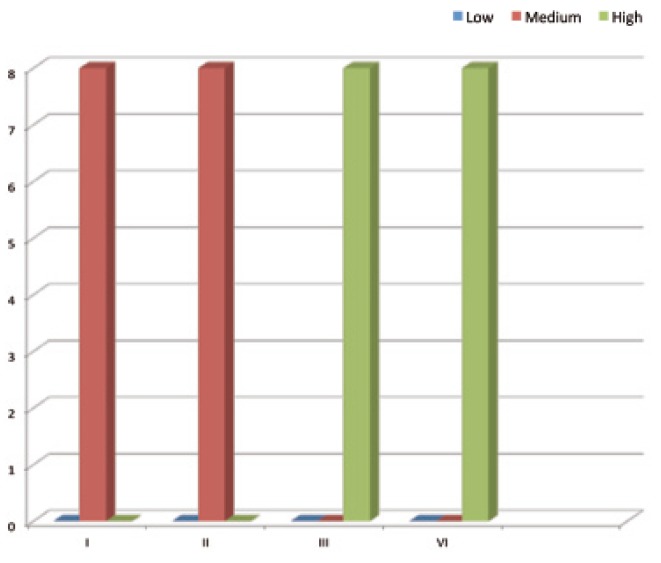
Adhesion test of Candida albicans with different types of orthodontic appliance.

The adhesion test, which is classified into low, medium and high, showed that the adhesion of *S. mutans*, using Chi-Square is low with type I and II, medium with type III and high with type IV in 100% of the sample used with a high significant differences (P< 0.001), (Fig. 1). On the other hand, Chi-Square for the Candida albicans adhesion test showed that it is medium with both type I and II and high with both type III and IV with a high significant differences (P< 0.001), (Fig. 2).

## Discussion

Knowledge of the growth and adhesion of cariogenic streptococci and *candida albicans* to orthodontic materials will highlight a better way of preventing enamel demineralization and white spot formation. This study will provide a primary step in identifying a mean to interfere with the process of growth and adhesion of pathogenic bacteria to the pellicle or plaque on the orthodontic appliances.

Direct comparisons of results with different studies must take into consideration the different methodologies used to examine this interaction between bacteria and hard surface. The most important factor that may explain the differences of the present study with the previous studies is the combination of bracket, arch wire and elastic together to simulate the fixed orthodontic appliance inside the patient mouth, which may provide more retentive surface for the formation of dental plaque.

The antimicrobial effect of materials was also examined. Manufacturers usually provide information about the physical properties of the materials, but often fail to include information about their antimicrobial properties (1).

In this study a significant difference in the inhibition zones formed with four types of the appliances used with both *S. mutans and Candida albicans*, the highest inhibition zone was found with type I, followed by type II then type III and the lowest with type IV, this mean that type I which include no metal in its component can inhibit the bacteria and yeast more than type II which has metal arch wire which in turn inhibit bacteria and yeast more than type III that has metal bracket which inhibit bacteria more than type IV that has metal bracket and arch wire.

On the other hand, after 48 hr incubation period, the inhibition zone formed with types I and II were decreased whereas those with types III and IV were increased, this mean that the effect of less metal components orthodontic appliance (type I and II) on the microbial inhibition was reduced with time in contrast to those with more metal components (type III and IV) showed more microbial inhibition with time. This could be related to the effect of components of sapphire brackets in types I and II and material coating the arch wire in type I.

The inhibition zone findings can explain the results of the adhesion test with different appliance types, which showed that the highest adhesion occurred with type IV followed by type III then type II and I, this mean that the weakest appliance in inhibiting the microorganisms (type IV) showed the highest bacterial adherence, whereas the strongest appliance in inhibiting microorganisms (type I) showed the lowest bacterial adhesion. Also the same adhesion pattern occurred with *Candida albicans* but with more adherences giving medium score in types I and II, and high score in types III and IV.

The differences in the bacterial adhesion amount can be explained by the difference in the surface characteristics of each material, including the surface roughness. Since the monocrystalline sapphire brackets (used in types I and II) had smoother surfaces than metal brackets (used in types III and IV) this could be confirmed by the findings of Lee et al. (19) who found smoother surface of sapphire than metal bracket in their study, therefore more bacterial adhesion was seen with type III and the most with type IV and this disagree with the findings of Fournier et al. (10) , who found that the affinity of microorganism for metal brackets was significantly lower than that for brackets made of plastic or porcelain, others like Papaioannou et al. (20) found that there is no significant difference in the adhesion of *S. mutans* to stainless steel, plastic and ceramic orthodontic brackets, but they used only brackets not as appliance sample used in the present study. Also Eliades et al. (8) found more microorganism attachment to mellatic brackets than others, whereas Gastel et al. (7) found less adhesion of microorganisms to metal bracket than others, but they used ceramic brackets so their results cannot compared directly with this study.

Yeast, *Candida albican*s species, was analyzed in this study because it is the most frequently found microorganism in infections of buccal mucosa. In this study *Candida albicans* showed more adherence than *S. mutans* with all types of appliances used, and its adherence to metallic appliances is higher than those with esthetic appliances and this disagree with the findings of Brusca et al. (1) who found metallic brackets decrease yeast adherence and composite brackets facilitated it.

This study finds that:

The capacity of microorganisms to adhere and grow is dependent on the materials of the orthodontic appliance used.

Appliance with high esthetic appearance, sapphire brackets and coated arch wire, showed the least adherence of *S. mutans and Candida albicans* in comparison to other appliances with less esthetic and more metal components.
